# Exosomal survivin facilitates vesicle internalization

**DOI:** 10.18632/oncotarget.26182

**Published:** 2018-10-09

**Authors:** Amber Gonda, Janviere Kabagwira, Girish N. Senthil, Heather R. Ferguson Bennit, Jonathan W. Neidigh, Salma Khan, Nathan R. Wall

**Affiliations:** ^1^ Center for Health Disparities Research and Molecular Medicine, Loma Linda University, Loma Linda, California, 92350, USA; ^2^ Department of Basic Sciences, Division of Biochemistry, Loma Linda University, Loma Linda, California, 92350, USA; ^3^ Department of Basic Sciences, Division of Anatomy, Loma Linda University, Loma Linda, California, 92350, USA

**Keywords:** survivin, membrane receptors, exosome, transferrin, cancer targets

## Abstract

Survivin, a member of the inhibitor of apoptosis (IAP) protein family plays a significant role in cell fate and function. It is significantly overexpressed in tumor cells and has been identified in most cancer cell types. A novel extracellular population has recently been identified and its function is still unknown. Emerging evidence continues to shed light on the important role the tumor microenvironment (TME) has on tumor survival and progression. This new population of survivin has been seen to enhance the tumor phenotype when internalized by recipient cells. In this paper, we sought to better understand the mechanism by which survivin is taken up by cancer cells and the possible role it plays in this phenomenon. We isolated the exosomal carriers of extracellular survivin and using a lipophilic stain, PKH67, we tracked their uptake with immunofluorescence and flow cytometry. We found that by blocking exosomal survivin, exosome internalization is reduced, signifying a novel function for this protein. We also discovered that the common membrane receptors, transferrin receptor, endothelin B receptor, insulin receptor alpha, and membrane glucocorticoid receptor all facilitate exosomal internalization. This understanding further clarifies the protein-protein interactions in the TME that may influence tumor progression and identifies additional potential chemotherapeutic targets.

## INTRODUCTION

Survivin is a multifunctional protein integral to tumor development and progression. It is the smallest member of the inhibitor of apoptosis (IAP) family containing only one of the baculovirus IAP repeat domains characteristic of this protein family [1]. Survivin plays an integral role in cell division as part of the chromosomal passenger complex, as well as in inhibiting the caspase dependent apoptosis pathway. These crucial cellular functions, including survivin’s role in the stress response [2], make it a key protein in cancer development and progression. Survivin is regulated by many established tumor suppressor proteins and consequently, in most cancer types (which often result from alterations of these tumor suppressors) it is overexpressed [3]. Additionally, increased levels of survivin are correlated with poor clinical outcomes [4]. As integrated as the protein is in the cancer cell, targeting it with cancer therapies appears promising. Several chemotherapeutics, including YM155 and LY2181308 [5–8], as well as certain vaccines [9] have been used successfully *in vitro* and are now being evaluated in clinical trials.

Survivin’s multiple functions have been closely tied with subcellular localization [10]. Nuclear survivin is primarily involved in mitosis [2, 11–13]. Cytoplasmic survivin plays a role in inhibiting apoptosis [10] and as a temporary location in the movement between nucleus and mitochondria [14]. Survivin’s primary role in apoptosis, however, is carried out by its mitochondrial localization. Here the protein interacts with SMAC and other apoptosis proteins [15]. Evidence has emerged illustrating that beyond Survivin’s intracellular pro-cancer functions, an extracellular population exists and plays a role in enhancing pathology [16–18]. When extracellular survivin is introduced to other cancer cells, proliferation increases and the rate of apoptosis decreases, even in the presence of radiation and chemotherapeutics [16]. In characterizing this extracellular survivin, it was discovered that it is localized to secreted vesicles called exosomes [19–21].

The understanding of the role extracellular vesicles, and exosomes in particular, play in the tumor microenvironment has grown exponentially in the last few years. Meehan and Vella recently outlined how integral exosomes are to the hallmarks of cancer described by Hanahan and Weinberg [22, 23], such as sustaining proliferative signaling and resisting cell death [16, 24]. Exosomes are small nanovesicles, 30-150nm in size, formed through the endocytic pathway. They have a lipid bilayer membrane that mimics the surface of the cell of origin. Due to the formation process, however, certain intracellular lipids and proteins are expressed on the extracellular surface of exosomes, such as phosphatidylserine [25, 26], heat shock proteins [27] and survivin [19, 21]. These external structures play a key role in exosome communication. Phosphatidylserine on the surface of a cell membrane is a signal to phagocytic cells that a cell is undergoing apoptosis and should be eliminated. Its presence on the exosome has been shown to similarly influence the uptake of these vesicles [25]. This internalization is a major mechanism of exosome influence on the tumor microenvironment. Genetic exchange occurs as exosomes carry functional mRNA and miRNA from cell to cell, altering the genetic and epigenetic makeup of the recipient cell [28], as seen in tumors [29], immune cells [30] and stem cells [31]. Internalization mechanisms of exosomes are prime targets for either reducing exosome uptake and/or preventing the distribution of the oncogenic loads; or as an opportunity to manipulate the natural exosome journey by mimicking the process with nanoparticles or modified exosomes containing pharmaceuticals for a more accurate delivery.

Uptake of these vesicles has been linked to many different cellular processes and has been well reviewed [32]. Phagocytosis, macropinocytosis, and various endocytosis methods have been identified, with endocytosis being perhaps the most common. Macropinocytosis is a non-specific sampling of the extracellular environment and can be argued to uptake exosomes by chance; a process by which microglia internalize exosomes [33]. Phagocytosis and endocytosis often involve receptors and protein interactions in order to internalize specific molecules. A few proteins have been proposed as key players in this process such as heparan sulfate proteoglycan on glioblastoma cells [34]. Other proteins that have been described as mediators in exosome uptake include integrins [35, 36], lectins [37–40], and tetraspanins [25, 41]. These proteins have been identified in interactions between the cell and the exosome in order to promote internalization. In this paper, we propose additional protein interactions that facilitate exosome uptake. We found a novel function for survivin as a mediator of exosome internalization as well as other common cellular receptors such as transferrin receptor, insulin receptor alpha, and endothelin B receptor to contribute to the uptake of exosomes in HeLa cells.

## RESULTS

### Vesicles isolated from HeLaS cell conditioned medium were defined as exosomes

**Figure 1:**
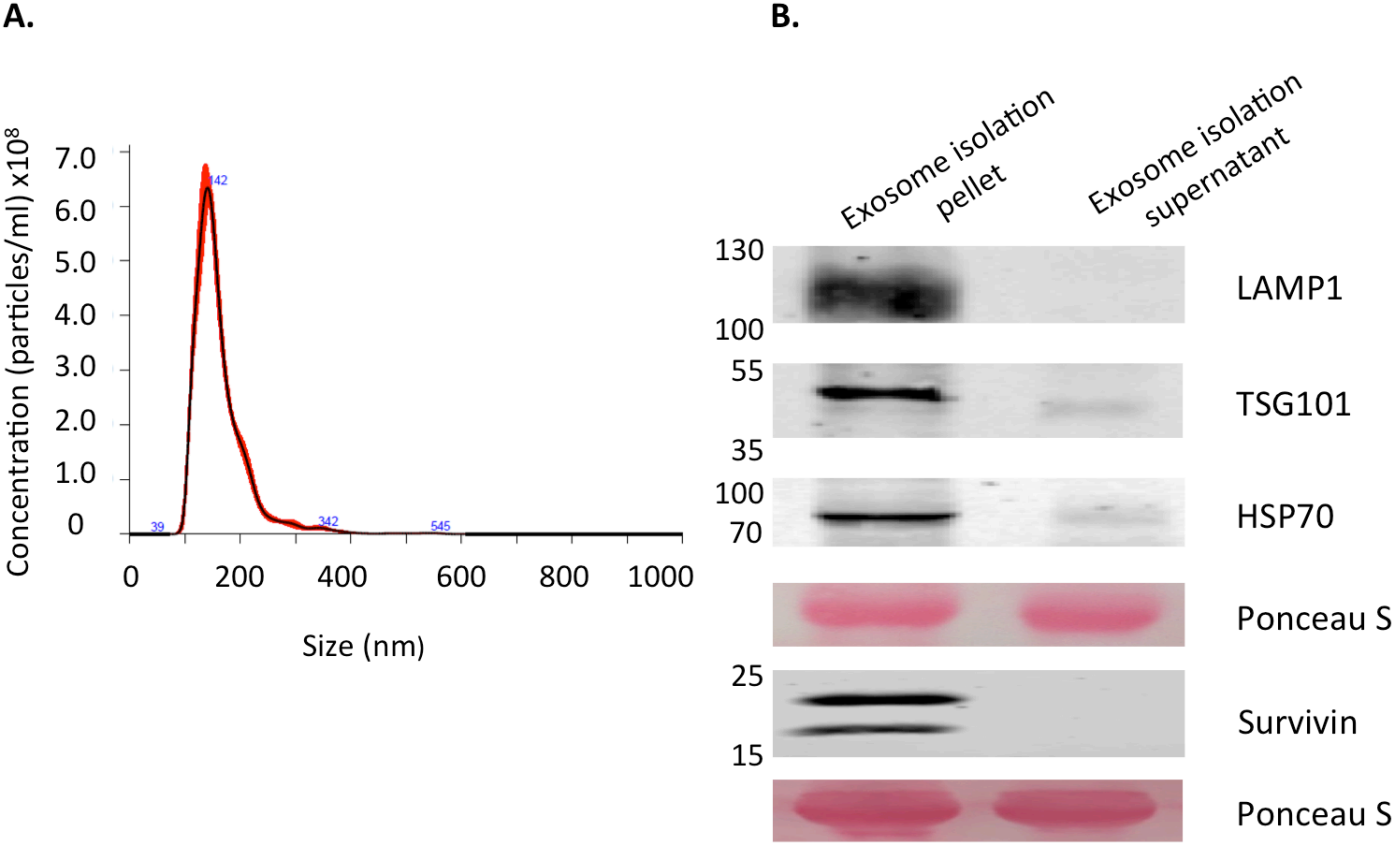
Characterization of exosomes derived from HeLaS cells **(A)** Extracellular vesicle size and concentration as measured using the Nanosight NS300. Mode size was within the exosome size range (30-150nm) at 142.5nm. Mean size was 162.7nm and concentration was 4.51x10^10^ particles/ml (±1.39x10^10^). **(B)** Western blot analysis of isolated vesicles and the corresponding vesicle-free supernatant. LAMP1, TSG101, and HSP70 were used as positive markers for exosome identification. The double band on the Survivin blot corresponds to the endogenous size as well as the HA/FLAG tagged Survivin expressed by the cells from which the exosomes originated. Ponceau S stain was used to verify equal amounts of protein were loaded. Both Nanosight readings and western blot data are representative of several repeated experiments.

Exosomes are just one of the vesicle types released from cancer cells. In order to isolate this particular population and separate it from the larger apoptotic bodies and microvesicles, conditioned medium (CM) was subjected to serial centrifugations, filtration, and ultracentrifugation over a sucrose cushion. The exosome-enriched population was measured by nanoparticle tracking analysis to determine both vesicle size and concentration [42]. Vesicles from this isolation process were identified to be within the accepted size range for exosomes, ranging from 100nm to 150nm with an average size of 131nm after repeated measurements (Figure 1A). Further classification of these vesicles as exosomes was determined by the presence of proteins enriched in exosomes (TSG101, LAMP1, and HSP70) [43, 44] (Figure 1B). Furthermore, these exosomes derived from a modified HeLa cell line with a Flag/HA tagged survivin, showed the presence of an 18.5kDa survivin protein in addition to the normal 16.5kDa size present in all cancer cells, as detected by Western blot (Figure 1B). This second band is absent on normal HeLa cells that lack the tagged survivin (data not shown). These exosomes were then used to treat the normal HeLa cell line.

### Exosome internalization depends upon surface proteins

**Figure 2:**
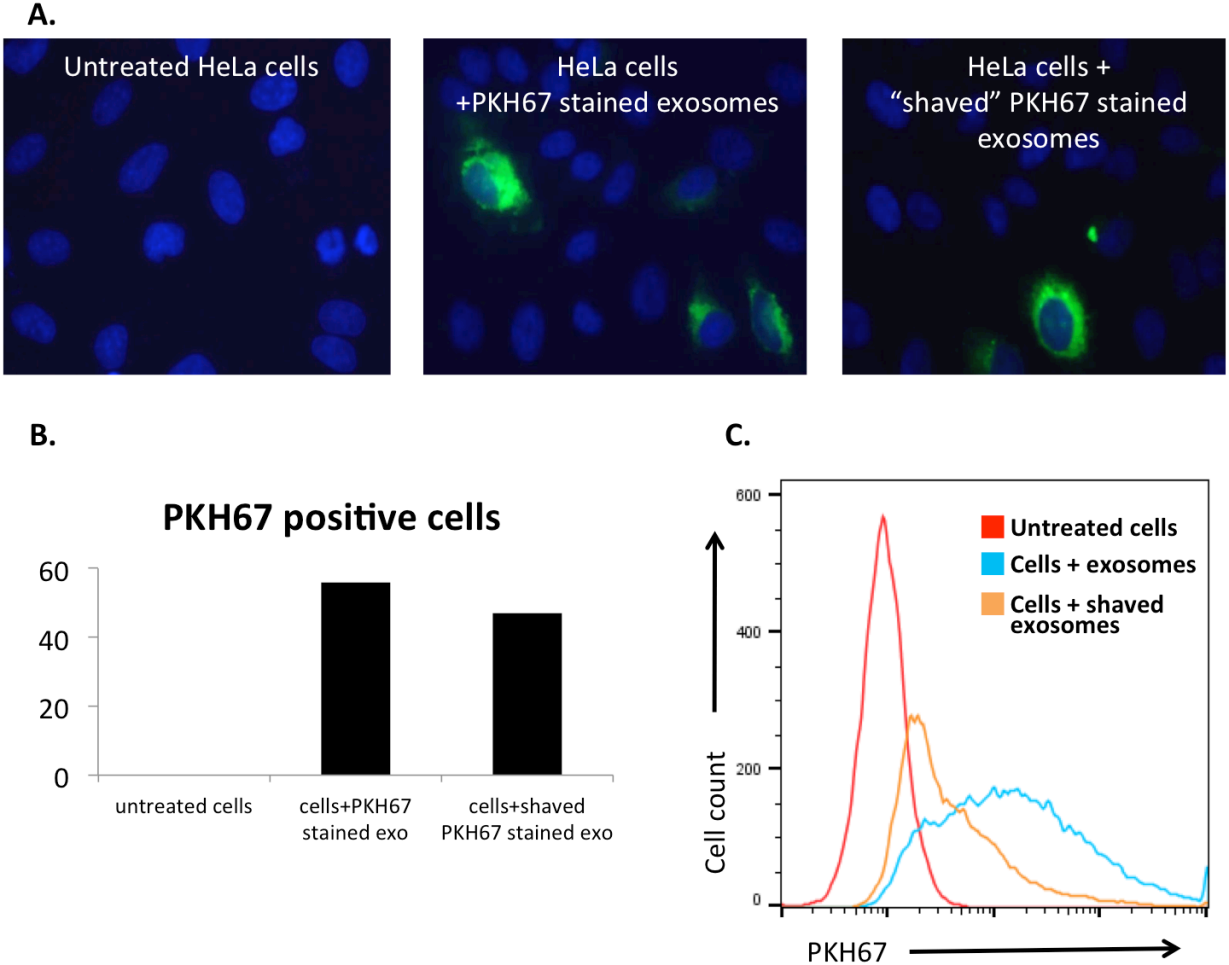
Exosome surface proteins are integral to internalization mechanisms Exosomal surface proteins were digested by proteinase K (“shaving”) and then introduced to cell culture. **(A)** Untreated and treated HeLa cells fixed and imaged with fluorescent microscopy (original magnification 40x; blue, DAPI stained nuclei; green, PKH67 stained exosomes.) Proteinase K treatment did not affect the PKH67 staining of exosomal membranes. **(B)** Cell count using the fluorescent imaging as described above. Cells staining green were counted as PKH67 positive cells, showing a higher percentage of internalization of intact exosomes than “shaved” exosomes. Total cell number was comparative between groups. **(C)** PKH67 labelled “shaved” exosomes were internalized at a lower rate than intact exosomes as shown by flow cytometry.

**Figure 3:**
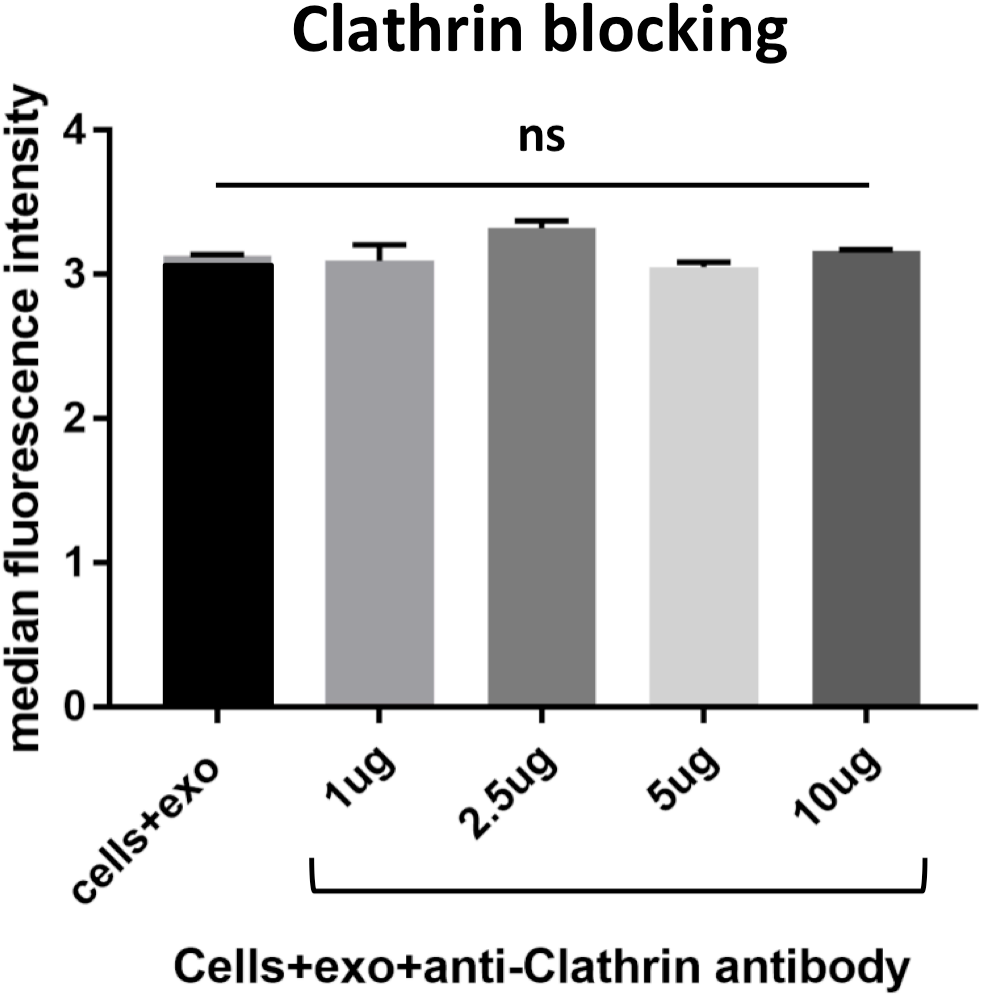
Blocking various membrane receptors changes the amount of exosome internalization by HeLa cells As a control, cells were incubated with an antibody to clathrin, an intracellular protein involved in endocytosis. As expected due to the inability of the antibody to cross the membrane barrier, no change was measured in the uptake of PKH67 stained exosomes by flow cytometry. Data is representative of 3 or more experiments. Experiments were analyzed on flow cytometry and data is presented with median fluorescence intensity (MFI). Significance was determined by student’s t-test or one-way ANOVA with ad hoc Tukey’s multiple comparison’s tests ^*^p<0.05, ^**^p<0.01, ^***^p<0.005, ^****^p<0.001.

**Figure 4:**
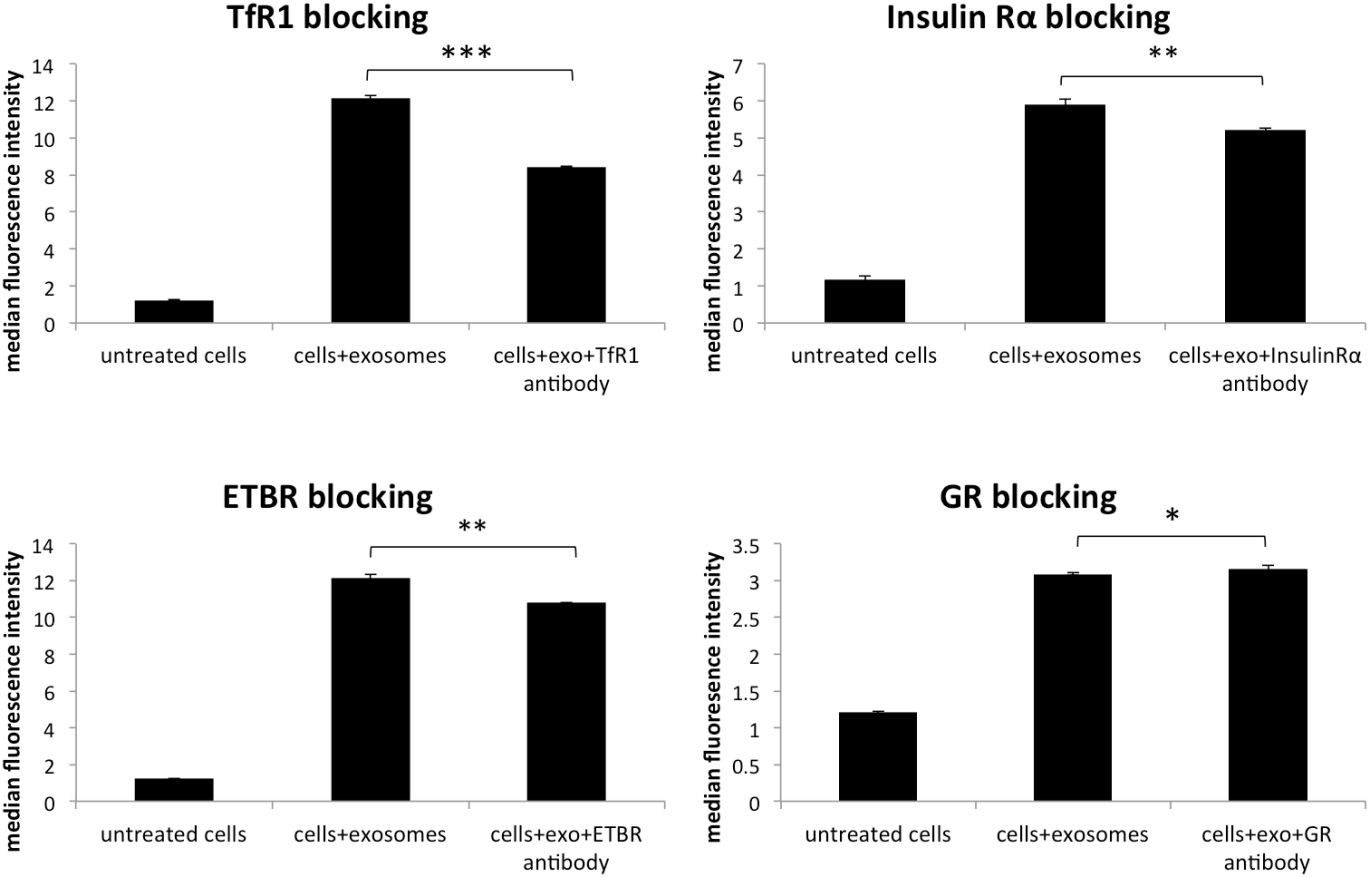
Blocking various membrane receptors changes the amount of exosome internalization by HeLa cells Reduction of PKH67 stained exosome internalization following antibody blocking of transferrin receptor 1 (TfR1), glucocorticoid receptor (GR), endothelin B receptor (ETBR), and insulin receptor alpha (IRα). Data is representative of 3 or more experiments. Experiments were analyzed on flow cytometry and data is presented with median fluorescence intensity (MFI). Significance was determined by student’s t-test or one-way ANOVA with ad hoc Tukey’s multiple comparison’s tests ^*^p<0.05, ^**^p<0.01, ^***^p<0.005, ^****^p<0.001.

**Figure 5:**
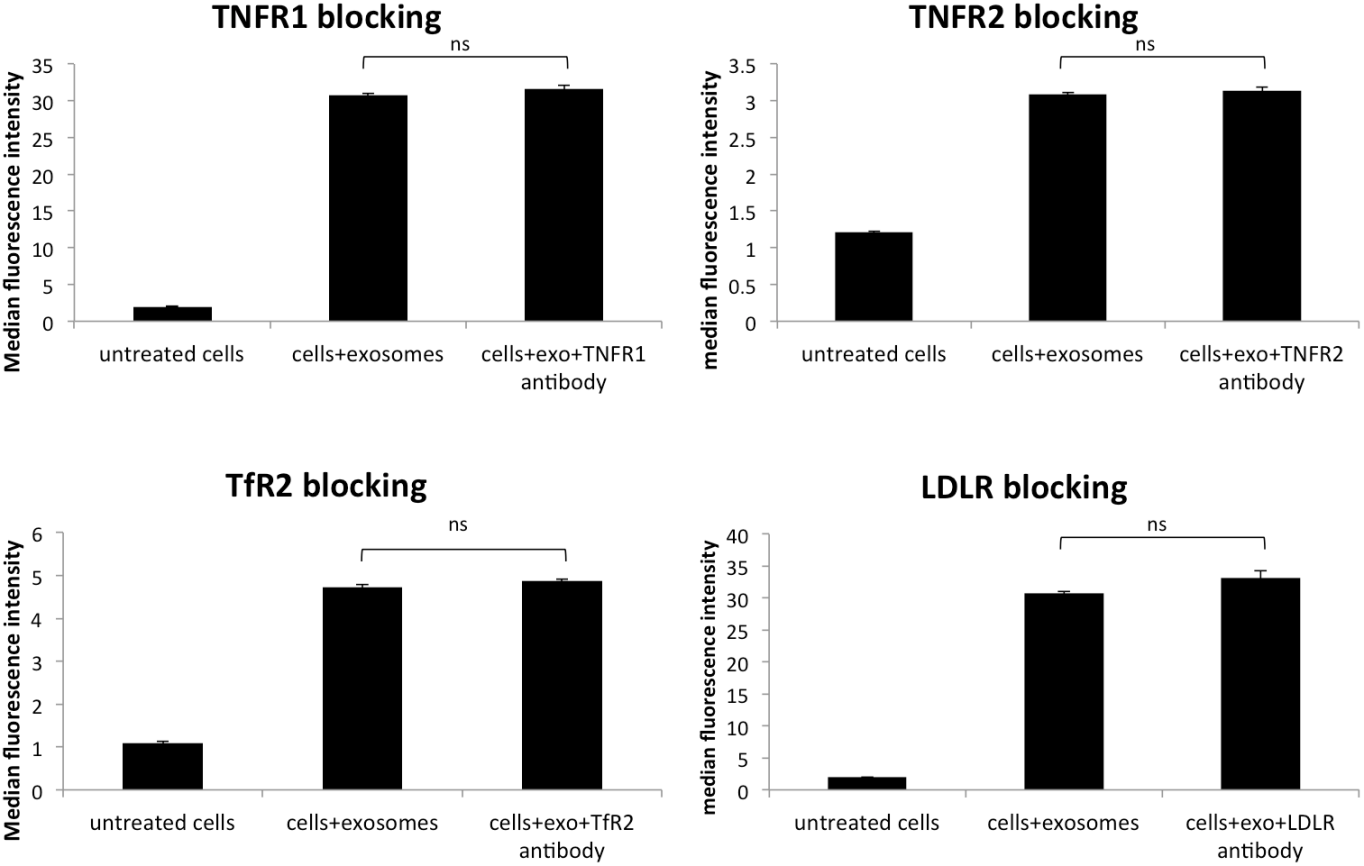
Blocking various membrane receptors changes the amount of exosome internalization by HeLa cells No change in PKH67 stained exosome internalization following antibody blocking of TfR2, tumor necrosis factor receptors (TNFR1 and TNFR2), and low-density lipoprotein receptor (LDLR). Data is representative of 3 or more experiments. Experiments were analyzed on flow cytometry and data is presented with median fluorescence intensity (MFI). Significance was determined by student’s t-test or one-way ANOVA with ad hoc Tukey’s multiple comparison’s tests ^*^p<0.05, ^**^p<0.01, ^***^p<0.005, ^****^p<0.001.

**Figure 6:**
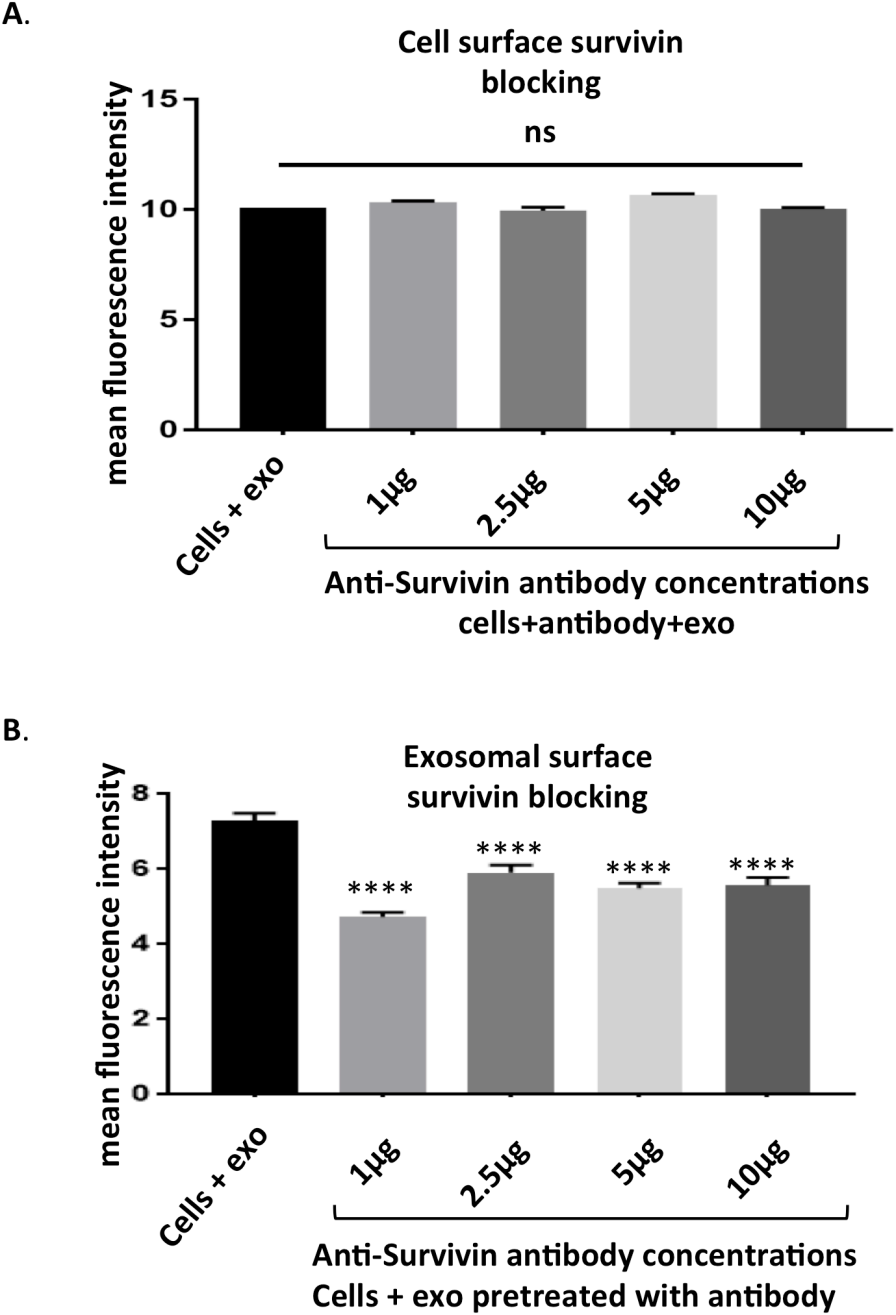
Survivin plays a role in exosome internalization **(A)** HeLa cells pre-treated with antibodies to Survivin showed no change in uptake of PKH67 stained exosomes as assessed with flow cytometry. **(B)** PKH67 stained exosomes pre-incubated with Survivin antibody show a decreased amount of internalization by HeLa cells. Data is representative of 3 independent experiments. Significance was determined by one-way ANOVA with ad hoc Tukey’s multiple comparison’s tests ^*^p<0.05, ^**^p<0.01, ^***^p<0.005 , ^****^p<0.001.

**Figure 7:**
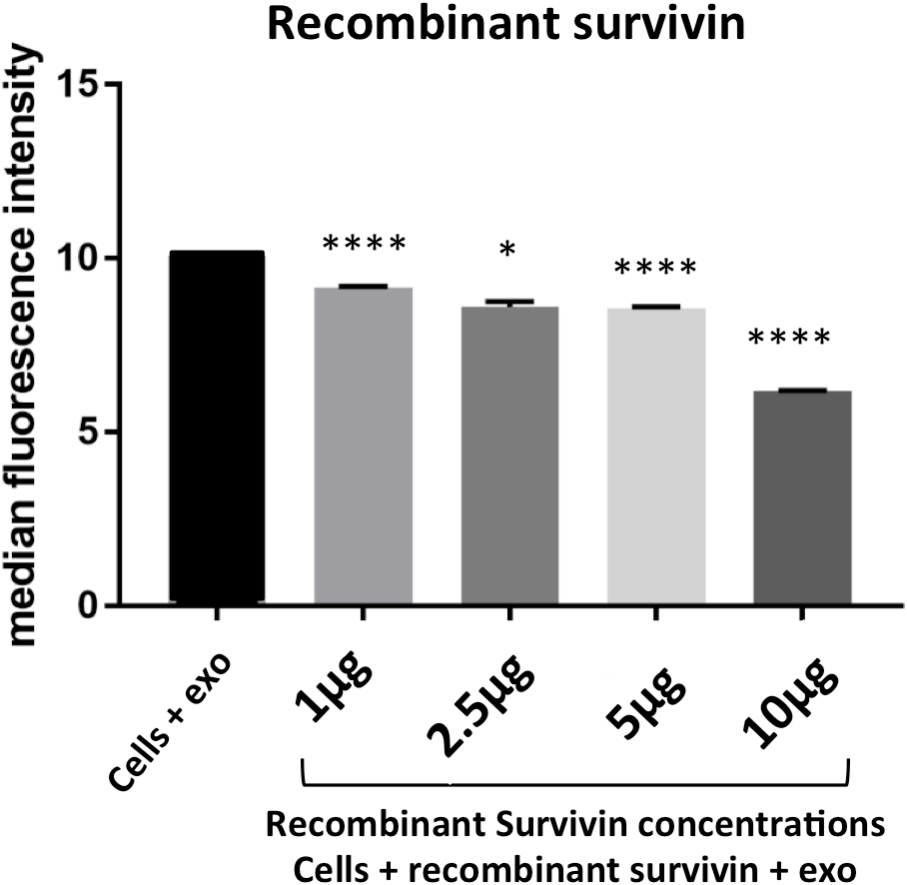
Survivin plays a role in exosome internalization HeLa cells co-incubated with PKH67 stained exosomes and soluble Survivin show a significant reduction in exosome uptake in a dose dependent manner. Data is representative of 3 independent experiments. Significance was determined by one-way ANOVA with ad hoc Tukey’s multiple comparison’s tests ^*^p<0.05, ^**^p<0.01, ^***^p<0.005, ^****^p<0.001.

In order to determine the ability of HeLa cells to internalize the isolated vesicle population, exosomes were stained with PKH67, a lipophilic fluorescent dye used regularly for exosome uptake tracking [25, 45, 46]. Exosomes were then re-assessed for size and concentration and no alteration to size was observed (data not shown). Exosome uptake has been described to take as little as 1 hour up to 12 hours, with a general plateau effect at 4 hours [39, 47–49]. HeLa cells were incubated with these exosomes for 4 hours and then analyzed with flow cytometry or with fluorescent microscopy (Figures 2-7).

The importance of exosome surface proteins in the internalization process was first assessed by degradation of these proteins with proteinase K, a serine protease. These “shaved” exosomes were then stained with PKH67 and assessed for internalization. Due to the lipophilic nature of the dye, PKH67 staining is not dependent on the presence of exosomal surface proteins, as fluorescent microscopy showed similar patterns of exosomal uptake in the presence of “shaved” exosomes and intact exosomes (Figure 2A). However, the significance of surface proteins in exosome uptake is shown with immunofluorescence (Figure 2B) and flow cytometry (Figure 2C), as the degree of internalization is significantly reduced when these proteins are degraded.

### HeLa cells take up exosomal survivin by receptor-mediated endocytosis

To further understand the role that membrane proteins play in the internalization of survivin, various cellular receptors were investigated. In tandem affinity purification (TAP), survivin was found to interact with transferrin receptor (TfR) and tumor necrosis factor receptor, (TNFR), as well as clathrin (data not shown). Using antibodies to block these receptors and others, we identified multiple receptors that play a role in the uptake of survivin containing exosomes. Blocking clathrin, an intracellular protein without extracellular presence, did not result in a change in uptake of exosomes (Figure 3). Instead, in the presence of increasing concentrations of TfR antibody, there was a reduction in the number of exosomes taken up by the cells (Figure 4). Using the same blocking strategy, both TNFR1 and TNFR2 antibodies were unable to significantly change overall exosome uptake (Figure 5), indicating a lack of contribution to the internalization mechanism. Four other cellular membrane receptors were similarly assessed to determine if these receptors were specific targets of exosomes. Membrane receptors, representative of various receptor families, were chosen as targets. Insulin receptor alpha (IRα), a tyrosine kinase receptor; endothelin B receptor (ETBR), a G-protein coupled receptor; and glucocorticoid receptor (GR), a primarily intracellular receptor; all showed similar trends to TfR1. Blocking these receptors with antibodies decreased the internalization of exosomes (Figure 4). Low-density lipoprotein receptor (LDLR), another classic endocytosis receptor, and transferrin receptor 2 (TfR2) showed a similar trend to that recorded with the TNFRs, indicating a minimal or negative role in exosome internalization (Figure 5).

### Facilitation of exosomal internalization is a novel role for survivin

As survivin has been documented on the surface of the exosome [19], its potential to influence exosome internalization was next assessed. Cells were first incubated with anti-survivin antibody and no change in uptake of exosomes was recorded across a 10-fold range of concentrations (Figure 6A). Next, exosomal survivin’s presence resulting in exosome internalization was tested. Stained exosomes were incubated with varying concentrations of the same antibody and then introduced to HeLa cells. The antibody was able to decrease the internalization of exosomes in all concentrations tested (Figure 6B). To further test survivin’s contribution to exosome internalization, soluble recombinant survivin was incubated with PKH67 stained exosomes and cellular uptake was again assessed using flow cytometry. At all concentrations of the recombinant survivin, uptake was diminished, but at the highest concentration (10 μg) of the recombinant protein, exosome uptake was significantly decreased (Figure 7), signifying that soluble survivin was competing with the exosomal population to be internalized by the cell.

## DISCUSSION

One of the crucial functions of the tumor microenvironment (TME) is to facilitate intercellular communication. This relay of information is greatly enhanced by the presence of extracellular vesicles, exosomes specifically, as conveyors of oncoproteins and oncogenes. Exosome trafficking in the TME is an essential aspect of tumor progression and has been established as a potential mechanism for the development of metastases [29, 43]. The protein interactions that facilitate the exosome-cell interaction are extensive and have been addressed in a number of recent reviews [32, 50–52]. In cancer progression, these interactions are crucial targets of exosomes containing cancer specific proteins. Here we affirm the necessity of such interactions to the internalization of these exosomes. When exosome surface proteins are degraded, uptake significantly decreases indicating that there are proteins on exosomes that are crucial to this process (Figure 2). One possible contributor is the anti-apoptotic protein survivin which is a protein ubiquitously overexpressed in cancer, carried by these vesicles and internalized by various cancer cell types. This particular protein has been shown to enhance the aggressiveness of the recipient cancer cell [16]. Thus determining the specific uptake mechanism would improve therapeutic targeting to include this intercellular communication.

Survivin has been extensively studied in the cell and its role in cell division and cell death are well established. However, its extracellular function is still under investigation. As a traditionally intracellular protein, extracellular interactions have not been well defined. Few groups have looked at this novel population, but these researchers have found evidence that there is more to the survivin story than its intracellular roles. Initial and subsequent reports of this population correlate its presence with poor clinical outcomes and enhanced pathology in cancer [16, 53, 54] and in arthritis [17, 55–57]. Other functions of this survivin population include modification of leukocyte function [18, 58], reduction of left ventricular myocyte apoptosis and dysfunction [59], and aiding in the protection of hyperglycemia-trigged cardiac cell death [60].

With its increasingly accepted role outside of the cell, the question has emerged as to how survivin is secreted. Two papers recently published have proposed a role for a membrane bound population [61, 62]. However, another theory that has been shown by our group and others is survivin’s release in exosomes [19, 21, 54, 60]. Other intracellular proteins without secretory peptides, like the heat shock proteins, have been found in the extracellular environment, often linked to exosomes, [27, 60]. Here for the first time, we show that exosomal survivin is not just a passenger, but plays an active role in the uptake of its carrier vesicle (Figure 6B). The presence of survivin on the surface of the exosome was unexpected, as it has not been previously identified on the surface of the cell. However, evidence is emerging in the extracellular vesicle field that some intracellular proteins are present on the exposed surface of the exosome [63]. Adding this function to this tumor-specific protein’s repertoire posits exciting new ways to target the tumor cell and its spread. With the evidence of exosomes targeting specific recipient cells, this could be a mechanism of cancer progression and metastasis, utilizing survivin’s unique overexpression.

In addition to survivin’s role in exosomal internalization, we explored the cellular contribution to exosome uptake. We identified four receptors, representative of different receptor families, locations, or functions and chosen for their overexpression on multiple cancer cells. Insulin receptor alpha (IRα) is a receptor tyrosine kinase, endothelin B receptor (ETBR) is a G-protein coupled receptor, glucocorticoid receptor (GR) is primarily a cytosolic and nuclear receptor, and low-density lipoprotein receptor (LDLR) is a classic endocytosis receptor. Four additional receptors were analyzed based on their interactions with survivin as discovered by tandem affinity purification/mass spectrometry/co-immunoprecipitation assays (data not shown): transferrin receptor (TfR1 and TfR2) and tumor necrosis factor receptor (TNFR1 and TNFR2).

Antibody blocking of IRα, TfR1, ETBR, and GR all showed a significant reduction in exosome internalization. This is a novel finding for each of these receptors. IRα and ETBR are both involved with ligand endocytosis [64–67] and have been shown to bind to multiple ligands [64, 68, 69]. This variation in ligand binding increases the likelihood of exosomes, which present a wide variety of surface proteins, using this route into the cell. Additionally, natural ligands of IRα have been identified on the surface of exosomes [70, 71]. The involvement of the GR in exosome uptake was unexpected, as it is primarily a cytosolic or nuclear receptor. However, data continues to accumulate identifying a population of GR at the surface of cells [72], which is being credited with the rapid non-genomic cellular responses to glucocorticoids frequently observed [73]. While membrane-bound GR has not yet been observed as an endocytic receptor, there is evidence of other steroid receptors (the estrogen receptor) that are located on the cell membrane and participate in ligand internalization [74]. Another receptor that was analyzed, TfR1, is a key endocytic receptor [75–77]. TfR1 preferentially binds transferrin but has been shown to bind to various other proteins, facilitating internalization of viruses and chemotherapeutic agent-bound antibodies [78, 79]. This, in addition to the presence of transferrin and transferrin receptor on cancer derived exosomes [70, 80], supports the hypothesis that TfR1 plays a role in exosome internalization. Our data presents this novel function of the receptor, revealing further potential in utilizing it in cancer therapeutics. Additional research is needed to identify the binding proteins on the exosome surface for each of these receptors.

While blocking TfR1 readily reduced exosome uptake, the interference with TfR2 did not have a similar effect. These two receptors share 45% homology but have significant differences in function and expression [81]. TfR2 expression in healthy adults is highly localized to the liver, but during development and in cancer, its expression is much broader [82], including HeLa cells as we saw in our lab (data not shown). Calzolari et al., has even identified TfR2 on exosomes [81]. Despite its presence on cancer cells, this receptor does not appear to be operating as an exosome transporter. Continued study is ongoing in our laboratory to determine if exosomal TfR2 plays a role in internalization. In addition to TfR2, the tumor necrosis factor receptors (TNFR1 and TNFR2) also failed to show a significant contribution to exosome internalization. The primary ligand for both receptors, TNFα, has been identified as exosome cargo [83, 84], but just as with TfR2, the presence of the ligand by itself is not enough to see a significant change when the receptor is blocked. Hawari et al., presented a possible explanation that TNFR signaling is regulated by shedding of the membrane protein through exosomes to compete with membrane bound receptors for its ligand [85]. Antibody blocking of the TNFR may have stimulated receptor shedding, increasing the competition for the exosome bound ligand, thus showing little change in exosome uptake. This same phenomenon is also a possible explanation for why blocking LDLR also had little effect on exosome uptake. LDLR, like the previously mentioned receptors, is prevalent in many different cancer cell types and is connected to poor prognosis [86–88]. Marzolo et al., studied megalin, a member of the LDLR family, and suggested the shedding of the receptor as a regulatory mechanism on exosomes could affect the availability of the ligand [89]. While these experiments showed no effect on exosome internalization, additional studies are needed to determine if a more indirect role in exosome trafficking exists.

Overall, we were able to establish the presence of novel protein interactions that facilitate the internalization of exosomes by cancer cells. None of the receptors identified reduced the uptake of exosomes completely, indicating that multiple internalization mechanisms are involved, as described by many other researchers. Several of these receptors are already being targeted in the clinic, such as the transferrin receptor, illustrating the fact that many of the current therapeutic methods may have broader effects than originally anticipated. With this increased understanding of exosome internalization, these receptors may be used in the development of targeted delivery of drug infused exosomes or nanoparticles. The disease-associated specificity of insulin and endothelin receptor overexpression may soon prove as successful a target as the transferrin receptor is proving to be with antibody based therapies. Additionally, the nanovesicle potential may present an alternative to the antibody approach for the transferrin receptor. The novel functions attributed to survivin will not only further advance the basic biologic understanding of this protein, but may also contribute to enhanced therapeutic approaches in the near future.

## MATERIALS AND METHODS

### Cell lines and antibodies

The cervical cancer cell line, HeLa, was obtained from American Type Culture Collection (ATCC, Manassas, VA). Cervical cancer cells capable of being grown in suspension (HeLaS) were a kind gift of Dr. Yang Shi at Harvard Medical School. These cells were used to generate cells with a Flag/HA tagged survivin as described previously [16]. These HeLaS pOZN WT survivin cells were those from which exosomes were isolated. All cells were maintained in DMEM (Cellgro, Mediatech, Inc., Manassas, VA) supplemented with 10% heat inactivated fetal bovine serum, 100 units of penicillin, 100ug/mL of streptomycin, and 100ug/mL L-glutamine (Corning, Mediatech, Inc, Manassas, VA). Cells were cultured at 37°C in a humidified incubator with 5% CO_2_ and grown to 60-70% confluency before treatment. For competition studies, full-length recombinant human survivin protein was obtained from Abcam (Cambridge MA).

Antibodies used in this paper are as follows: mouse monoclonal anti-CD71 (transferrin receptor 1-TfR1), mouse monoclonal anti-transferrin receptor 2 (TfR2) mouse monoclonal anti-tumor necrosis factor receptor 1 (TNFR1), mouse monoclonal anti-tumor necrosis factor receptor 2 (TNFR2), rabbit polyclonal anti-insulin receptor α, mouse monoclonal anti-low density lipoprotein receptor (LDLR), rabbit polyclonal anti-endothelin B receptor (ETBR), mouse monoclonal anti-glucocorticoid receptor (GR), mouse monoclonal anti-tumor suppressor gene 101 (TSG101), mouse monoclonal anti-clathrin (Santa Cruz Biotechnology, Santa Cruz, CA), rabbit monoclonal anti-β actin (Li-Cor, Lincoln, NE), purified anti-human CD107a (LAMP-1) (Biolegend, San Diego CA), mouse monoclonal anti-HSP70 antibody, and polyclonal anti-survivin antibody (Novus Biologicals, Littleton, CO).

### Exosome isolation and characterization

Exosomes were isolated from HeLaS pOZN WT Survivin cells using differential centrifugation. Cells were grown to 60-70% confluency and media was aspirated. Cells were washed with 1XPBS and incubated for 24 hours with exosome-depleted FBS/DMEM. Depletion of exosomes from FBS was performed as follows: 20% heat-inactivated FBS/DMEM was centrifuged for 16 hours at 100,000xg, then completed to have a final medium of 10% heat-inactivated FBS, 100 units of penicillin, 100ug/mL of streptomycin, and 100ug/mL L-glutamine. The depleted medium from the 24-hour incubation, or conditioned medium (CM), was then collected and serially centrifuged. Dead cells, non-cellular debris, and larger vesicles were eliminated after 3 centrifugations: 10min at 400xg, 20min at 2000xg, and 30min at 10,000xg on a Beckman Allegra X-15R centrifuge (SX4750A rotor) and ThermoScientific Sorvall Legend X1R centrifuge (F15-8X50Y). This CM was then subjected to ultracentrifugation on a Beckman Coulter XL-90 ultracentrifuge in order to pellet and isolate exosomes. CM was spun for 2 hours at 100,000xg on an SW27 rotor and then filtered through a 0.22μm filter and placed on a 30% sucrose cushion. This was then spun for 180,000xg for 3 hours on an SW41 rotor and washed with 1xPBS for 2 hours at the same speed. Pelleted vesicles were then analyzed for exosome characteristics. Real-time imaging, size, and concentration were assessed using nanoparticle-tracking analysis on the NanoSight NS300 (Malvern Instruments, Malvern, UK) following the manufacturer’s protocols. Briefly, vesicles isolated were diluted 1:100 in filtered 1xPBS, sonicated for 30 seconds to reduce aggregation and loaded on to the machine with a syringe pump. Machine settings were as follows: software-NanoSight NTA 3.2, camera level-13-14, detection threshold-5, capture time-60sec, captures-5, flow rate-30.

### Expression plasmid and generation of stable cell lines

The detailed procedure for cloning and propagation has been described previously [90, 91]. In brief, recombinant retroviruses expressing a bicistronic messenger RNA containing open reading frames of Flag-HA (hemagglutinin)-tagged human survivin and interleukin-2 receptor (IL-2R)-α was constructed and transduced into HeLa cells. The infected HeLa cells were sorted by anti-IL-2R monoclonal antibody (mAb) conjugated with magnetic beads, and the resulting Flag-HA-Survivin stable cell line was propagated as a suspension culture (HeLaS POZn WT survivin). The expression level of wild type (WT) survivin was evaluated by Western analysis and immunohistochemistry with anti-Flag and HA antibodies (Santa Cruz Biotechnology, Inc., Santa Cruz, CA).

### In-gel trypsin digestion and MS

Proteins (50 μg) were resolved on a 4-12% gradient gel after which gels were stained using Coomassie. Protein bands were excised manually and washed with 50% (v/v) methanol and 5% (v/v) acetic acid. The gel pieces were then dehydrated in acetonitrile and dried in a SpeedVac concentrator (Savant, Farmingdale, NY). Proteins were reduced using 10mM dithiothreitol (DTT) in 100 mM ammonium bicarbonate for 30 min at room temperature. The DTT solution was removed and the proteins were alkylated for 30 min at room temperature using 100mM iodoacetamide after which the gel pieces were dehydrated as before. Gel pieces were rehydrated in 100 mM ammonium bicarbonate and then dehydrated and dried as previously described. Proteins were tryptically digested using MS grade trypsin (Promega, Madison, WI), added at a final concentration of 20 ng/mL to fully cover the gel pieces. Digestion was performed at 37°C overnight. Peptides were recovered with 30 μl, 50% (v/v) acetonitrile and 5% (v/v) formic acid twice. All supernatants were pooled and dried in a SpeedVac concentrator for 1 h.

Tryptic peptides were analyzed on our ThermoFinnigan LCQ Deca XP system that includes a surveyor HPLC and a PicoView 500 (New Objective, Woburn, MA) for performing nanoflow electrospray ionization. The ml/min. flow of the surveyor HPLC pump was split to achieve a 200 – 300 nanoliter/min flow exiting a PicoFrit column (New Objective) packed with BioBasic C18 beads (10 cm, 5 μm, 300 Å). Samples were loaded onto a Michrom Bioresources (Auburn, CA) cap-trap at 5 μl/minute and washed with mobile phase A (aqueous 2% acetonitrile with 0.1% formic acid). Peptides were then eluted onto the column and into the mass spectrometer using a gradient of 0-75% mobile phase B (aqueous 90% acetonitrile with 0.1% formic acid). The mass spectra acquisition was operated in the data dependent mode with one MS scan (300 – 1500 m/z) and three MS/MS scans of the most intense ions in the MS scan.

We used the Sequest algorithm implemented on the TurboSequest software package to identify proteins based on the MS/MS spectra. The resulting Sequest hits were filtered based on the charge state and Xcorr value to require Xcorr >= 1.5, 2.0, and 2.5 for single, double, and triple charged ions, respectively.

The MS/MS fragmentation spectra were searched against a current human protein database (March 2009) containing 37,391 reference sequences. The search algorithms Sequest [92], Mascot [93], and X! TANDEM [94], were used to identify peptides and proteins. The significance of identified peptides and proteins were determined using the PeptideProphet [95] and ProteinProphet [96], respectively, algorithms as implemented in Scaffold 2 (Proteome Software, Portland, OR). We included only peptides with a Scaffold score of ≥ 95% (5% false discovery rate) in the results.

### Immunoblot analysis

Western blot was used to determine the presence of exosome-enriched proteins on the vesicle population, survivin presence, and receptor presence. After harvesting cells, lysates were prepared using a lysis buffer composed of 50mM Tris-HCL pH 7.5, 1% Triton-X, 0.25% deoxycholic acid, 150nM sodium chloride, 1mM sodium orthovanadate, 20mM sodium fluoride, 0.2mM ethylene glycol tetraacetic acid, 1mM ethylenediaminetetraacetic acid, 1mM phenylmethylsulfonyl fluoride, 1x protease inhibitor cocktail (Roche Life Science, Indianapolis, IN), sonicated, and briefly heated to 95°C. To remove lipid contamination, samples were centrifuged at 13,000 rpm for 1 minute and the pellet discarded. This step was not performed with exosome lysis. Protein concentration was determined using the Pierce BCA protein assay (ThermoScientific, Waltham, MA) according to the manufacturer’s protocol. Proteins (30-50ug) were heated to 95°C for 5 minutes and fractionated using 10%, 12%, or 15% Bis-Tris polyacrylamide gels. Proteins were transferred to nitrocellulose membranes (BioRad, Hercules, CA) and blocked for 1 hour at room temperature in 5% milk (w/v in 1xPBS-0.1%Tween). Subsequently, membranes were incubated overnight at 4°C in primary antibody. Membranes were washed with 1xPBS-Tween20 then probed for 1 hour at room temperature with goat anti-rabbit or goat anti-mouse DyLight 800 conjugated secondary antibodies (ThermoScientific, Grand Island, NY). Membranes were then washed and imaged using the Odyssey infrared imaging system (Li-Cor, Lincoln, NE) and analysis with the Image Studio version 5.2 software. β-actin was used as a loading control for cellular assays and Ponceau S stain was used for exosome loading controls. Data are representative of multiple independent experiments.

### Exosome processing

HeLaS pOZN WT survivin-derived exosomes were labeled with PKH67, a lipophilic dye, according to manufacturer’s protocol (Sigma Aldrich, St. Louis, Missouri) with the following modifications. Exosomes were incubated in diluent C and PKH67 for 5minutes at room temperature. Exosome-depleted media was then added to stop the absorption of the dye and exosomes were centrifuged at 180,000xg for 1.5 hours using an SW41 rotor followed by centrifugation with the same settings in 1xPBS. Pellets were then resuspended and analyzed on the Nanosight NS300 for concentration and size.

For exosome “shaving”, prior to the PKH67 staining, exosomes were incubated in 100ug/mL proteinase K for 30 minutes. In order to stop the protein degradation, equal amounts of 5X protease inhibitor (Roche, Indianapolis, IN) were added for 10 minutes at room temperature. Protease digested exosomes were then centrifuged at 100,000xg for 1 hour in a Type 65 rotor with 1xPBS. The supernatant was aspirated, exosomes labeled with PKH67, and analyzed as described above.

### Antibody blocking

In preparation for antibody and exosome treatment, Hela cells were grown to 60-70% confluency in a 12 well plate and washed with 1xPBS. Cells were then incubated in exosome-depleted medium with individual antibodies at a concentration of 10ug/well (6.6ug/ml) unless stated otherwise. Other concentrations used were 1ug/well (0.6ug/ml), 2.5ug/well (1.6ug/ml), and 5ug/well (3.3ug/ml). Cells were incubated with antibodies for 30 min at 4°C and the unbound antibodies were washed off with 1xPBS. Negative controls (untreated cells) and positive controls (cells plus exosomes) were incubated in depleted media alone. Cells were then incubated with 0.8x10^9^ particles/mL exosomes for a period of 4 hours at 37°C. Negative controls were incubated with depleted media alone and positive controls were treated with the same concentration of exosomes. Cells were harvested and plated in a 96-well plate for flow cytometry analysis.

### Flow cytometry

Cells were washed with 1xPBS and incubated for 30min in 7AAD viability dye. Cells were then washed and fixed in 1% paraformaldehyde and examined on the MacsQuant Analyzer 10 (Miltenyi Biotec) and analyzed using FlowJo software version 10. Antibody blocking of the exosomes was completed in a similar fashion with the exception that exosomes instead of cells were incubated with the antibody for 30 min at 4°C and then added to the cells for the 4 hour incubation. This process was the same for all antibodies.

### Fluorescent microscopy

HeLa cells were cultured on coverslips in 6 well plates and grown to a confluency of 60-70% before treatment. Antibody and exosome treatments were performed as described above. After the 4-hour incubation with exosomes, cells were washed with 1xPBS and incubated in 3.7% formaldehyde for 20 minutes at room temperature. Formaldehyde was then washed off with 3 successive 1xPBS washings and the coverslip was affixed to glass slides using Vector Vectashield mounting medium for fluorescence with DAPI (Vector Laboratories, Burlingame, CA). Images were obtained using an Olympus BX50F-3 fluorescent microscope (Olympus Scientific Solutions Americas Corporation, Waltham, MA) using 20x and 40x lenses and imaging on FITC (PKH67) and DAPI (mounting media with DAPI) channels. Phase contrast images with fluorescent PKH67 stained exosomes were imaged on the Keyence BZ-X700 all-in-one fluorescent microscope (Keyence Corporation, Itasca, IL). Cells were cultured as described previously and treated with PKH67-stained exosomes for 4 hours. The cells were then imaged live with this microscope.

### Statistical analysis

One-way ANOVA with an ad hoc Tukey’s multiple comparisons test or Student’s paired one-tailed t-test was performed on these experiments. Statistical analyses were performed using Graph Pad Prism 7.04 for Windows (GraphPad Software, La Jolla, CA) software and Microsoft Excel 2013 data analysis software. A probability of less than 0.05 was considered to be significant. Data are presented as the mean and standard deviation and are representative of at least 3 independent experiments.
